# Root System Architecture, Copper Uptake and Tissue Distribution in Soybean (*Glycine max* (L.) Merr.) Grown in Copper Oxide Nanoparticle (CuONP)-Amended Soil and Implications for Human Nutrition

**DOI:** 10.3390/plants9101326

**Published:** 2020-10-08

**Authors:** Elham Yusefi-Tanha, Sina Fallah, Ali Rostamnejadi, Lok Raj Pokhrel

**Affiliations:** 1 Department of Agronomy, Faculty of Agriculture, Shahrekord University, Shahr-e Kord, Iran; yusefi@stu.sku.ac.ir; 2 Department of Electroceramics and Electrical Engineering, Malek Ashtar University of Technology, Tehran, Iran; rostamnejadi@mut-es.ac.ir; 3 Department of Public Health, The Brody School of Medicine and Department of Health Education and Promotion, College of Health and Human Performance, East Carolina University, Greenville, NC 27834, USA

**Keywords:** metal oxide nanoparticles, bioaccumulation, recommended dietary allowances, daily values, essential nutrients

## Abstract

Understanding the potential uptake and biodistribution of engineered nanoparticles (ENPs) in soil-grown plants is imperative for realistic toxicity and risk assessment considering the oral intake of edibles by humans. Herein, growing N-fixing symbiont (*Bradyrhizobium japonicum*) inoculated soybean (*Glycine max* (L.) Merr.) for a full lifecycle of 120 days, we assessed the potential influence of particle size (25, 50, and 250 nm) and concentration (0, 50, 100, 200, and 500 mg/kg soil) of Copper oxide nanoparticles (CuONPs) on: (1) root system architecture, (2) soil physicochemical attributes at the soil–root interface, and (3) Cu transport and accumulation in root, stem, leaf, and seed in soybean, and compared them with the soluble Cu^2+^ ions and water-only controls. Finally, we performed a comparative assessment of total seed Cu levels in soybean with other valuable food sources for Cu intake and discussed potential human health implications. Results showed particle size- and concentration-dependent influence of CuONPs on Cu uptake and distribution in root, stem, leaf, and seed. Alterations in root architecture (root biomass, length, volume, and area) were dependent on the Cu compound types, Cu concentrations, and their interactions. Concentration–response relationships for all three sizes of CuONPs and Cu^2+^ ions were found to be linear. Furthermore, CuONPs and Cu^2+^ ions had inhibitory effects on root growth and development. Overall, soybean responses to the smallest size of CuONPs–25 nm—were greater for all parameters tested compared to the two larger-sized CuONPs (50 nm, 250 nm) or Cu^2+^ ions. Results suggest that minor changes in soil-root physicochemical attributes may not be a major driver for Cu uptake in soybean. Cu bioaccumulation followed the order: root > leaf > stem > seed. Despite reduction in root architecture and seed yield, the smallest size CuONPs–25 nm led to increased total seed Cu uptake compared to the larger-sized CuONPs or Cu^2+^ ions. Our findings also suggest that soil amendment with CuONPs, and more so with the smallest size of CuONPs–25 nm—could significantly improve seed nutritional Cu value in soybean as reflected by the % Daily Values (DV) and are rated “Good” to “Very Good” according to the “World’s Healthiest Foods” rating. However, until the potential toxicity and risk from CuONP-fortified soybean seed ingestion is characterized in humans, we caution recommending such seeds for daily human consumption when addressing food Cu-deficiency and associated diseases, globally.

## 1. Introduction

Soybean (*Glycine max* (L.) Merr.) is an important and economical legume cultivated worldwide for food and feed products [1,2]. Soybean seeds contain 20% oil [3], about 35–40% protein, and a complete set of essential amino acids [4] critical for improving human nutrition plan and heath [5]. Soybean is considered the best source of plant protein and a standard for other plant protein sources [6]. It also serves as an excellent source of carbohydrates (35%) and essential elements, including copper (Cu), zinc (Zn), calcium (Ca), magnesium (Mg), iron (Fe), manganese (Mn), and phosphorus (P), critical for human and animal nutrition purposes [6,7,8]. Furthermore, it contains metabolites, such as isoflavone, saponins, phytic acids, and oligosaccharides, which serve various biological functions [7,8]. Soybean, like other legumes, enables nitrogen fixation by establishing a symbiotic association with a specific rhizobium bacterium, *Bradyrhizobium japonicum* [9].

The world’s population is growing rapidly and is forecasted to reach 9.6 billion by 2050 [10], thereby increasing demand for agricultural production (70% for grain production by 2050) [11]. On the other hand, due to limited arable lands and usable water resources, applying high amounts of chemical fertilizers is a conventional approach to achieve increased food production and meet population demands, globally [12]. Crops, such as soybean, need nutrients to support growth and yield, and soil nutrient deficiency can significantly reduce N_2_-fixation, growth, and plant performance [13]. Macronutrients (N, P, K, Ca, Mg, and S) and micronutrients (Fe, Zn, Mn, and Cu) promote different morphological and physiological functions in plants, including enzyme activities and oxidation-reduction processes [13]. Micronutrients can be supplemented to plants using chemical fertilizers [14]. Interest in the use of nanofertilizers has recently increased due to their unique physicochemical characteristics, which are not found in their bulk or ionic counterparts [15,16,17,18]. Micronutrients, particularly in “nano” form, could improve both the yield and nutrient quality of crops compared to the micronutrients that are conventionally delivered in an ionic or a bulk form [19]. Nanofertilizers may come in different forms: (1) nanofertilizers made of macro-and micro-nutrients; (2) application of nanomaterials as a nutrient carrier [18]. Accordingly, the former could supplement one or more nutrients to plants, and the latter could enhance conventional fertilizer delivery efficiency but may not directly supply nutrients to plants [12]. Plant response to engineered nanoparticles (ENPs) depends on the chemistry of the soil and nanoparticle, the exposure dose, and the species of crop [20,21,22]. Upon soil application, ENPs can enter the root, then penetrate the cell wall/plasma membrane, reaching the root cortex and entering the xylem vessels, thereby moving upward through the stele to aerial plant parts [23,24].

Cu is a redox metal that can exist in the form of cupric (Cu^2+^/Cu (II)) and cuprous (Cu^+^/Cu (I)) ions [25]. Being anti-tumorigenic, Cu (II) elicits cytotoxicity through cellular apoptosis [26]. In plants, Cu is a cofactor in a variety of proteins, including in cytochrome C oxidase, plastocyanin, receptors of ethylene, and Cu/Zn-superoxide dismutase [27,28,29]. Cu-containing proteins are also involved in biological reactions, such as Fe oxidation [25], chlorophyll synthesis, and carbohydrate metabolism. About 70% of Cu is present in plant chloroplast [30]. Biological activities, including metabolism of cell wall and ethylene, photosynthesis, mitochondrial respiration, and protection against toxic free radicals and oxidative stress, are disrupted with Cu deficiency [27,28,29]. Moreover, Cu deficiency could affect overall plant growth and yield of fruit and seed [30]. Exposure to toxic levels of Cu could increase chlorosis, necrosis, root growth inhibition, and cause an increase in lignin content, leading to reduced cell expansion and nutrient uptake [13,31,32,33]. A mixture of ionic and nanoparticles of Cu, Zn, and B were investigated, and mixed responses were observed for growth, yield and nutrient uptake in soybean [34]. Likewise, several studies have reported mixed results for Cu-NPs in lettuce, wheat, and mung bean [35,36,37].

Many foods contain Cu naturally, and Cu is also available over the counter as a dietary supplement. Several cuproenzymes consist of Cu as a cofactor and play key roles in myriad of physiological and biochemical synthesis processes, including in iron metabolism, energy production, neuropeptide activation, and synthesis of neurotransmitters and connective tissues in humans [38,39,40,41]. Cu also influences neurohormone homeostasis, brain development, angiogenesis, pigmentation, and regulation of gene expression and immune functioning [41]. Superoxide dismutases that contain Cu play a major role in defense against oxidative damage [42,43]. According to the National Health and Nutrition Survey (NHANES) data from 2009–2012, 6% to 15% of adults aged 19 years and older who do not take dietary supplements containing Cu have a Cu intake below the Estimated Average Requirement (EAR; an average daily intake estimated to meet the requirements of 50% of healthy individuals) [44]. Of those adults who use supplements, 2.2% to 7.2% had an intake below the copper EAR [44]. It is known that Cu deficiency, although uncommon, could lead to anemia, hypopigmentation, connective tissue disorders, osteoporosis, hypercholesterolemia and other bone defects, ataxia, abnormal lipid metabolism, and increased risk of infection [41,45,46]. It is, therefore, crucial to find ways to improve Cu levels in edibles, such as soybean, that are inexpensive and consumed widely.

Because soil-applied ENPs will first come in contact with the root surfaces, following which biouptake and biodistribution of the elements (in pristine and/or modified form) could occur within the plant tissues, it is important to investigate the potential effects of ENPs on the root system architecture, soil–root interface, and potential accumulation in different tissues/organs in plants relative to its soluble ions, while building knowledge on ways to improve nutritional elements such as Cu in edible plant parts. Thus, in the present study, growing soybeans (*Glycine max* (L.) Merr.) for a full lifecycle of 120 days, we assessed potential influence of particle size (25, 50, and 250 nm) and concentration (0, 50, 100, 200, and 500 mg/kg soil) of Copper oxide nanoparticles (CuONPs) on: (1) root system architecture, (2) soil physicochemical attributes at the soil–root interface, and (3) Cu transport and accumulation in root, stem, leaf, and seed, and compared with soluble Cu^2+^ ions (as a positive control) and water-only controls. A comparative assessment of total seed Cu levels in soybean with chickpea and other valuable food sources for Cu intake and its implications to human health are also presented.

## 2. Material and Methods

### 2.1. CuONPs Synthesis, Characterization and Localization in Seed

We employed the sol-gel method to synthesize three different sized CuONPs (25 nm, 50 nm, and 250 nm). The details of the synthesis protocol were reported previously by our group [47]. Crystallinity (phase formation, crystal structure, microstructure) and particle size distribution of the NPs were characterized using X-ray diffraction (XRD) and field emission scanning electron microscopy (FE-SEM), respectively. Dynamic light scattering (DLS) allowed us to estimate the hydrodynamic diameter (HDD) and zeta potential of the CuONPs.

Potential NP localization in the seed embryo upon CuONP exposure was investigated using TEM (ZEISS EM LEO906) and compared with the control seed embryo samples. Samples were prefixed in 2.5% (*v*/*v*) glutaraldehyde for 1.5 h, washed 3 times in 0.1 M phosphate buffer, post-fixed in 1% osmium tetroxide for 1 h, dehydrated in acetone, and infiltrated and embedded in epoxy resin. Samples were then imaged with TEM and image analysis was performed using Digimizer.

### 2.2. Experimental Set Up, Soil Preparation and Exposure Conditions

Experiments were conducted at the Shahrekord University (50°49′ E, 32°21′ N), Iran, and followed a completely randomized design (CRD) with three replications per treatment. The treatments included: CuCl_2_ (Cu^2+^ ions; positive control), three different sizes of CuONPs (25, 50, and 250 nm) and five concentrations (0, 50, 100, 200, and 500 mg/kg soil) of CuONPs, or Cu^2+^ ions. Each experimental unit consisted of two plants for a total of six plants per treatment (n = 6).

Soil was collected from a 0–30 cm depth from the corn fields of the Shahrekord plain, air dried for 7 days, and sieved (2 mm pore size) to separate any larger soil aggregates, wood chips, and rocks. The soil constituted sand, silt, and clay at 16%, 58%, and 26%, respectively. Background Cu concentration of the soil was 0.538-mg/kg soil. Additional soil characteristics are presented in Table S1. Based on the soil test, fertilizers of urea (86 kg/ha, 46% N; as a starter) and triple superphosphate (100 kg/ha, 44% P_2_O_5_ equivalent to 19% P) were added to the soil before planting ensued. Soil pH and electrical conductivity (EC) were continuously measured until harvest (at intervals of 30 days), following García-Gómez et al. [48], in soil:water (1:5 suspensions). For soil amendment, copper compounds (Cu^2+^; CuONPs: 25 nm, 50 nm, and 250 nm) were suspended in 100 mL distilled water to achieve the desired nominal concentrations (0-, 50-, 100-, 200-, and 500-mg CuONP/kg soil or Cu^2+^/kg soil). Untreated soil represented the negative control, and dissolved Cu^2+^ ions were added to soil as a positive control. CuONPs and Cu^2+^ solutions were dispersed by ultrasonication (100 W, 40 kHz) for 30 min at 25 °C, following which the suspensions were stirred with a magnetic bar to further minimize aggregation before adding to the soil, then mixed with soil using a hand-mixer before sowing.

### 2.3. Planting and Crop Management

The present study was conducted under outdoor microcosm conditions mimicking experimental conditions in the natural field environment, with an average monthly temperature of 12.5 °C and annual precipitation of 314 mm. The growth climate is described as moderate and cold with a warm and dry summer. Seeds of soybean (Kowsar cultivar) were procured from the Seed and Plant Improvement Institute, Karaj, Iran. Plants were cultivated in polyethylene (PE) pots. Each pot contained 4 kg soil. For easier plant removal from the pot at harvest, each pot had an inner liner of PE mesh (with 50 holes of 5 mm diameter for drainage), which was filled with a layer of washed gravel (500 g). Before sowing, seeds were immersed in the suspension of nitrogen (N)-fixing symbiotic bacteria, *Bradyrhizobium japonicum*, for 30 min and two inoculated seeds were planted per pot at a soil-depth of 2.5 cm following soil-amendment with CuONPs or Cu^2+^ ions. Irrigation was based on field capacity. A sub-sample of water was evaluated for total Cu concentration using an inductively coupled plasma-optical emission spectroscopy (ICP-OES) for each irrigation episode. Upon maturity (120 days), whole plants were harvested; aerial parts and root were then separated, oven dried (70 °C for 48 h), weighed separately in paper bags, and stored in plastic bags until analysis. Seeds were air-dried and stored.

## 3. Measurement of Root Parameters

To characterize the root system, root length (RL), root volume (RV), root area (RA), and root density (RD) were measured. After washing the roots with distilled water, a 1000 mL graduated cylinder was used to determine the root volume based on water displacement. Root length was measured from the stem base to the longest root using a ruler. Root dry weight was measured using an analytical balance and expressed in grams per plant. Root density was expressed as a ratio of mean root dry weight to root volume [49]:

(1) $$RD=\frac{RDW}{RV}$$

where RDW is the mean root dry weight (g) and RV is the root volume (cm^3^).

Root area was calculated following the Equation [49]:

(2) $$RA\;=\;2{\left.\left(RL\;\times \;\pi \;\times \;RV\right.\right)}^{0.5}$$

where RA is the mean root area (cm^2^) and RL is the root length (cm).

### 3.1. ICP-OES Analysis of Total Copper Uptake

For the measurement of total Cu uptake or bioaccumulation in different plant organs (root, stem, leaf, and seed), the samples (0.3 g) were washed several times with deionized water and dried at 70 °C for 48 h. Subsequently, they were digested with 10 mL HNO_3_ (150 °C for 1 h), then with 2 mL HClO_4_ at 215 °C for 2 h (5:1 *v*/*v*). The digests were further diluted up to 10 mL using deionized water. The extracts were filtered before being analyzed using an ICP-OES (Varian Vista-Pro Axial) for total Cu concentrations in different plant parts [50]. Six-point calibration curves were derived using standards at 0.312, 0.625, 1.25, 2.5, 5.0, and 10 mg Cu/L. Blank constituted Milli-Q water with 2% HNO_3_. The limit of detection (LOD) was 30 µg Cu/L.

### 3.2. Seed Copper Concentration Comparison with Recommended Dietary Allowance (RDA) and Daily Value (DV)

We compared soybean seed Cu levels with those in chickpea seeds and other food sources. 100 g of soybean seeds is assumed to be equivalent to 100 g of chickpea seeds per serving of ½ cup, which is equivalent to 3.5 ounces. The National Academies of Sciences, Engineering and Medicine’s Food and Nutrition Board (FNB) has developed the Dietary Reference Intakes (DRIs) for Cu and other nutrients for human intake recommendations [51]. The DRI offers a set of reference values used for planning and assessing nutrient intakes for healthy people. Recommended Dietary Allowance (RDA) is an average daily intake considered sufficient to meet the nutrient requirements of (almost) all (97–98%) healthy individuals, typically employed in planning diets that are nutritionally adequate for individuals [51]. The U.S. Food and Drug Administration (FDA) has developed Daily Values (DVs) to assist consumers with comparing the nutrient contents in foods and dietary supplements within the context of a total diet. We used an RDA value of 0.9 mg (900 µg) for adults and children aged 4 years and older [52] to calculate % DV (equivalent to % RDA) for soybean seeds grown under CuONP or Cu^2+^ ion treatments. Foods providing 20% or more of the DV are considered to be high sources of a nutrient. Next, we compared various Cu food sources from the U.S. Department of Agriculture, Agricultural Research Service’s Food Data Central database [53]. Finally, we classified our soybean seed Cu data based on the “World’s Healthiest Foods” rating following the rule: Excellent, if DV ≥ 75%; Very Good, if DV ≥ 50%; Good, if DV ≥ 25% [54].

### 3.3. Statistical Analysis

Because the data satisfied normal probability distribution, all data were used untransformed unless stated otherwise. A two-way analysis of variance (ANOVA) was performed using SAS (SAS Inc., ver. 9.4) to determine significant differences in crop responses to different treatments following a completely randomized experimental design. A Fisher’s Least Significant Difference (LSD) test at the 0.05 probability level was performed to further compare the means between the treatment groups. We determined if the concentration–response curves were linear (monotonic) or nonlinear (nonmonotonic) by coupling visual inspection of the curves with a simple decision rule, as previously described in our companion paper (ref. [47]): based on the linear regression line, if the co-efficient of determination (R-squared) value is 65% or greater, the concentration–response curves were deemed linear, suggesting that the plant response changes linearly with the concentration applied following the relationship: y = ax + b, where y is dependent variable, x is independent variable, and a and b are model parameters. The R-squared values of the models are tabulated below.

## 4. Results and Discussion

### 4.1. Nanoparticle Characterization

**Figure 1:**
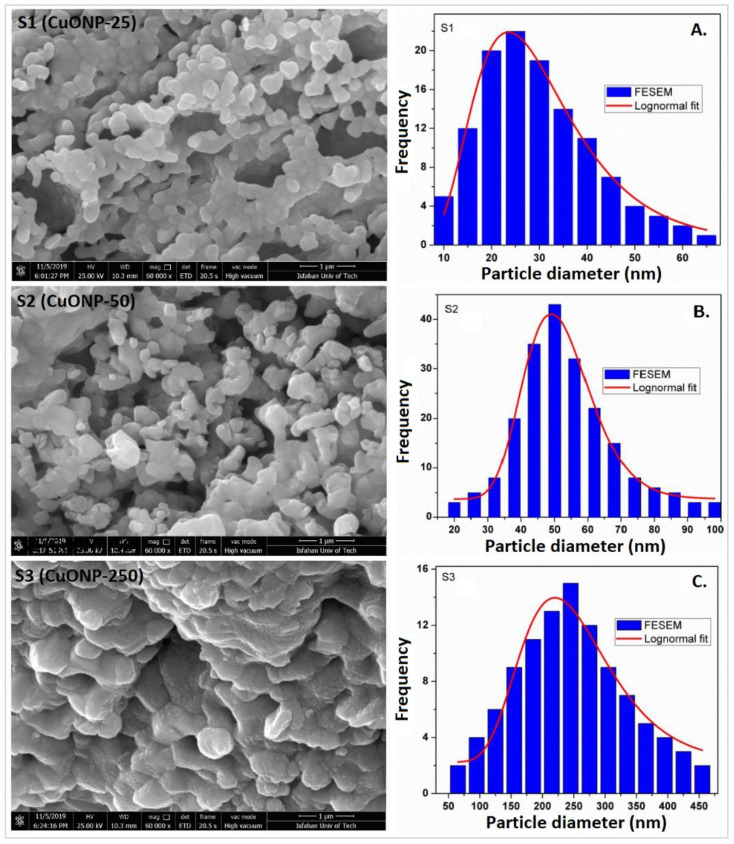
Electron micrographs (FE-SEM) of three different size CuONPs with particle size distribution (PSD): (**A**) S1 showing an average diameter of 25 nm, denoted CuONP–25; (**B**) S2 showing an average diameter of 50 nm, denoted CuONP–50; (**C**) S3 showing an average diameter of 250 nm, denoted CuONP–250.

**Figure 2:**
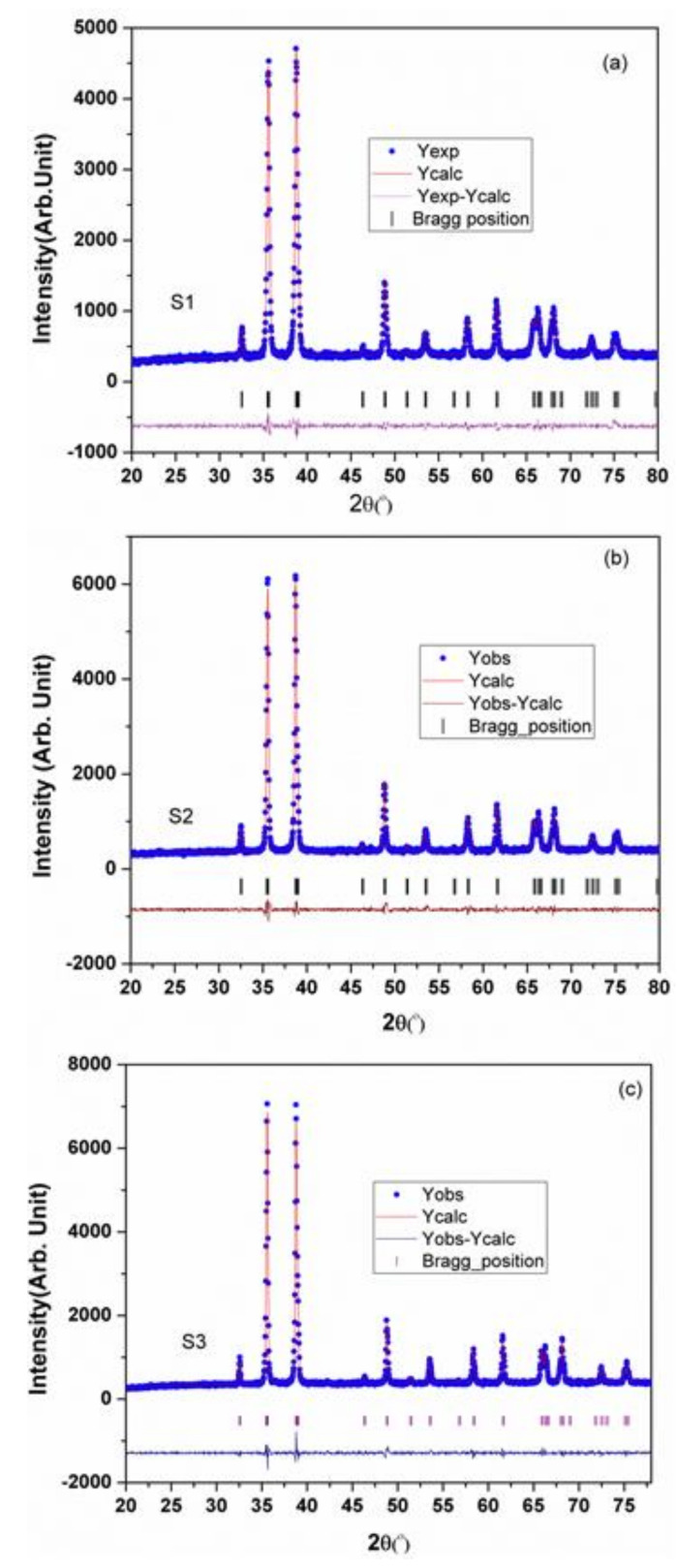
XRD patterns with Reitveld analysis for three different sized CuONPs: (**a**) S1 = CuONP–25 nm; (**b**) S2 = CuONP–50 nm; (**c**) S3 = CuONP–250 nm.

The lognormal fitting of particle size distributions from the FE-SEM micrographs revealed the mean particle size for S1, S2, and S3 samples as 25 nm, 50 nm, and 250 nm, respectively (Figure 1). The Reitveld analyses of the XRD patterns are presented in Figure 2 and the structural parameters obtained are shown in Table 1. The results indicate that the single-phased CuONPs are crystallized in monoclinic structure and are highly pure. All CuONP samples have high negative charges with similar zeta potentials (about −52 mV), but with different hydrodynamic diameters (189.0 nm, 195.1 nm, and 915.6 nm). These analyses were part of our previous companion paper [47].

#### 4.1.1. Root Dry Weight

The results showed significant effects of copper compound type (Cu_type_; *p* < 0.0001) and concentration (C; *p* < 0.0001), and their interactions (Cu_type_ × C) on root dry weight (*p* ≤ 0.01) (Table 2). Particle size- and concentration-dependent inhibition in root weight was observed upon exposure to CuONPs; the two forms of Cu (CuONPs and Cu^2+^) at all concentrations tested led to a decrease in root dry weight. However, the effect of CuONP–25 nm was significantly greater than the larger-sized CuONP or Cu^2+^ ion treatments (Table 3). Root dry weight decreased in a linear concentration-dependent manner for the larger-sized CuONPs (CuONP–50, CuONP–250) and Cu^2+^ ions, whereas for CuONP–25 nm, the relationships appeared nonlinear (Table 4). Although the lowest root dry weight occurred at 500 mg/kg CuONP–25 nm, it was not significantly different from the 200 mg/kg CuONP–25 nm and 500 mg/kg CuONP–50 nm treatments. Compared to the control, the root dry weight upon exposure to CuONP–25 nm was reduced by 44.6%, 59.6%, 71.9%, and 82.75% for 50, 100, 200, and 500 mg Cu/kg soil treatments, respectively (Table 3). The highest root dry weight (5.45 g/plant) was observed in control plants, and this did not significantly differ from 50 mg/kg Cu^2+^ treatment. In addition, unlike the smallest sized CuONP–25 nm, plants treated with the larger-sized CuONPs did not show significantly different root dry weights at all tested concentrations. Furthermore, the average root dry weight was not significantly different among CuONP–250 nm and Cu^2+^ ion treatments up to 100 mg/kg soil (Table 3).

Although at low concentrations, Cu serves as a necessary micronutrient for plant growth and development, higher concentrations at the soil–root interface could lead to harmful effects on plant growth [55]. Cu concentration in different plant tissues is typically in the range of 2.0–50 µg/g dry weight [56]. The findings of our study suggest particle size- and concentration-dependent toxicity of CuONPs in root biomass, and that CuONPs’ toxicity may not be related to Cu^2+^ ions released because Cu^2+^ ion-only treatments resulted in significantly lower toxicity compared to CuONP treatments at all concentrations (50–500 mg/kg) tested in soil-grown soybean (Table 3). Previously, it was documented that exposure to CuONPs (<50 nm) could decrease stem and root growth in rice [33], barley [57], and wheat [58]. Likewise, particle size- and concentration-dependent toxicity of CuONPs in *Arabidopsis* showed reduced root growth, root lignification, and plant biomass [59]. Additionally, root lignification and growth modification in *Glycine max* [60] and *A. thaliana* [31] were reported with Cu^2+^ exposure (0–5 µM), suggesting that the absorbed fraction of the dissolved Cu ions can lead to decreased root growth in soybeans.

#### 4.1.2. Root Length

ANOVA showed significant effects of Cu compound type (Cu_type_; *p* < 0.0001), copper concentration (C; *p* < 0.0001), and the interaction term Cu_type_ × C (*p* < 0.01) on soybean root length (Table 2). The results showed particle size- and concentration-dependent root length in soybeans upon exposure to CuONPs, and the CuONP–25 nm treatment led to shorter root length compared to the larger-sized CuONP and Cu^2+^ ion treatments at most concentrations tested (Table 3). The concentration–response curves for CuONPs and Cu^2+^ were observed to be linear (Table 4). Root length was, on average, two times lower upon 500 mg/kg CuONP–25 nm treatment compared to the control. Root length was not significantly different at all concentrations tested for CuONP–50 nm and CuONP–250 nm (Table 3). The effects of CuONP–250 nm at 50 mg/kg was not significantly different from 50 and 100 mg Cu^2+^/kg treatments and the control (*p* < 0.05).

The changes in plant root morphology upon exposure to CuONPs may point to a localized release of Cu in the form of NPs and/or ions upon contact between root cell surface and the NPs [61]. Released Cu can alter meristematic activity and epidermal cell differentiation into root hairs [61]. CuONPs (40–80 nm) were found to inhibit root elongation in maize (95.73%) and rice (97.28%) but at a higher concentration of 2000 mg/L [62]. Our results for root length are consistent with earlier studies that reported decreased root length in mustard [63], soybean [55], and mung bean upon exposure to CuONPs [64].

#### 4.1.3. Root Volume

Table 5 shows changes in root volume in soybean upon exposure to different Cu compounds. ANOVA showed that the effect of Cu compound type (Cu_type_; *p* < 0.0001), concentration (C; *p* < 0.0001), and the interaction term (Cu_type_ × C; *p* ≤ 0.05) were statistically significant for root volume in soil-grown soybean (Table 2). These results clearly show changes related to particle size and concentration in root volume upon exposure to CuONPs. Amongst all the treatments tested, root volume was significantly lowest at 500 mg/kg for CuONP–25 nm but was not significantly different between 200 mg/kg CuONP–25 nm and 500 mg/kg CuONP–50 nm treatments, similar to root dry weight (*p* < 0.05; Table 5). Furthermore, root volume for CuONP–250 nm was not significantly different from Cu^2+^ ion treatments at all concentrations. At 50 mg/kg Cu^2+^ ions, root volume was not significantly different from the control. For all CuONPs types, root volume was not significantly different at two lower concentrations (50 and 100 mg/kg) (Table 5). The observed decrease in root volume upon exposure to small-sized CuONPs at higher concentrations could be attributed to decreased cell division, lateral root count, and root elongation (Table 3). Consistent to our study, a previous study also found reduced cell division and cell elongation, leading to reduced root elongation in sand-grown wheat upon treatment with CuONPs (>10 mg Cu/kg) [61].

#### 4.1.4. Root Area

Our findings showed that root area was significantly affected by Cu compound type (Cu_type_; *p* < 0.0001), concentration (C; *p* < 0.0001), and the interaction term (Cu_type_ × C; *p* < 0.001) (Table 2). The results generally show particle size- and concentration-dependent changes in root area upon exposure to CuONPs, and the effects mirrored other root parameters (Table 3; Table 5) in that the decreasing effects of CuONP–25 nm were significantly greater compared to the larger-sized CuONPs or Cu^2+^ ions treatments for most concentrations tested (Table 5). The lowest root area was documented at 500 mg/kg CuONP–25 nm, which was not significantly different from CuONP–25 nm at 200 mg/kg (Table 5). Further, at 50 mg/kg Cu^2+^ ions, root area was not significantly different from control, akin to the root length and root volume (Table 3 and Table 5). Also, there was no significant difference in root area between CuONP–250 nm and Cu^2+^ ions at all concentrations tested (*p* < 0.05). Small-sized CuONPs at higher concentrations decreased root area significantly due to reduced root length (Table 3) and root volume (Table 5). For CuONP–250 nm, the trend of root area change was relatively minimal across different concentrations tested. Consistent with our results, previous studies have also shown that exposure to CuONPs could lead to decreased root growth in rice [33], barley [57], and wheat [58].

#### 4.1.5. Root Density

**Figure 3:**
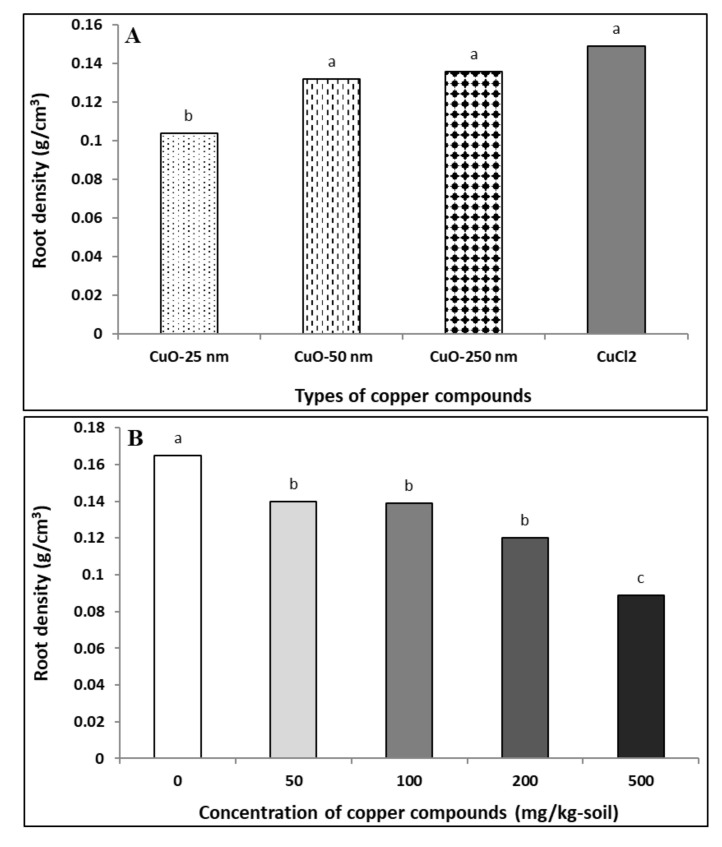
Effect of copper compound types (**A**) and concentrations (**B**) on soybean root density. The data for (**B**) are the aggregates of root density for all CuONPs and Cu^2+^ ions combined as they were not significantly different at the same concentration treatment. Same letter above the bars indicates no significant difference between the treatments according to the Fisher’s LSD test (*p* ≤ 0.05).

Root densities in soybeans exposed to different Cu compounds are presented in Figure 3A. The effects of Cu compound type (Cu_type_; *p* ≤ 0.0001) and concentration (C; *p* < 0.0001) were significant on root density, except for the interaction term (Cu_type_ × C; *p* > 0.5; Table 2). Soybeans exposed to CuONP–25 nm showed the lowest root density compared to the larger-sized CuONPs (50 and 250 nm) or Cu^2+^ ions. Additionally, root density was not significantly different between larger-sized CuONP (50 and 250 nm) and Cu^2+^ ion treatments (Figure 3A). At the highest concentration of Cu compounds (500 mg/kg), the root density was significantly reduced in comparison with the lower concentrations for all Cu compound types and the control (Figure 3B). Herein, CuONP–25 nm showed a significant decrease in root density with concomitant decrease in root dry weight (Table 3) and root volume (Table 5). In addition, with increasing concentration, the decrease in root density was greater compared to control (Figure 3B). In agreement with our findings, a previous study has documented particle size- and concentration-dependent toxicity of CuONPs in *Arabidopsis* and that root growth was reduced with CuONP treatments [59].

### 4.2. Root Copper Uptake

**Figure 4:**
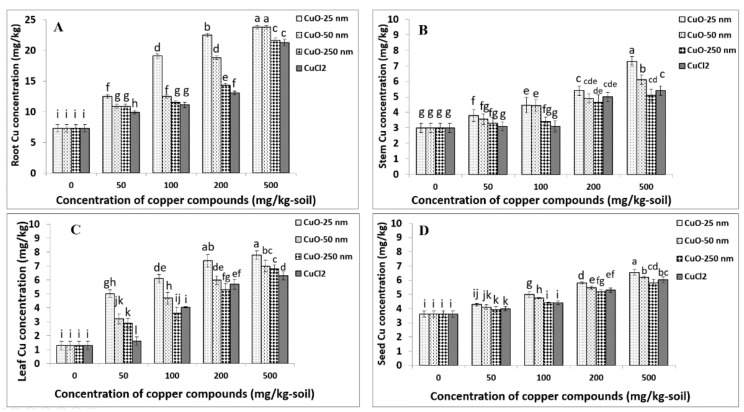
Cu uptake in soybean root (**A**), stem (**B**), leaf (**C**), and seed (**D**) upon exposure to soil amended with CuONP–25 nm, CuONP–50 nm, CuONP–250 nm, and CuCl_2_, at different concentrations. Same letter above the bars indicates no significant difference according to the Fisher’s LSD test at *p* ≤ 0.05.

ANOVA indicated that the effects of Cu compound type (Cu_type_), concentration (C), and the interaction term (Cu_type_ × C) were significant for Cu concentration in soybean root (*p* < 0.0001) (Table S2). The results show particle size- and concentration-dependent Cu uptake and accumulation in soybean roots upon exposure to CuONPs during the full lifecycle of 120 days. For all Cu compound types, root Cu concentrations significantly increased compared to the control, and the concentration–response curves for the larger-sized CuONPs (CuONP–50 nm, CuONP–250 nm) and Cu^2+^ ions were deemed linear (Figure 4A and Table 4). Cu uptake was found to be significantly greater for CuONP-25 nm compared to the larger-sized CuONP or Cu^2+^ ion treatments at all concentrations tested. Compared to control roots, the average Cu concentrations in plants treated with CuONP–25 nm at 50, 100, 200, and 500 mg/kg concentrations increased 1.7, 2.6, 3.0, and 3.3 times in soybean roots, respectively (Figure 4A). The highest Cu concentration in soybean root was observed for 500 mg/kg CuONP–25 nm treatment, and this was not significantly different from the CuONP–50 nm treatment at the same concentration. Likewise, Cu uptake from CuONP–25 nm treatment at 100 mg/kg was not significantly different from the 200 mg/kg CuONP–50 nm treatment (*p* < 0.05). Root Cu concentrations upon 50 mg/kg of CuONP–25 nm treatment were not significantly different from 100 mg/kg CuONP–50 nm and 200 mg/kg Cu^2+^ ion treatments. In addition, at 500 mg/kg, root Cu uptakes upon CuONP–250 nm and Cu^2+^ ion treatments were not statistically significant (*p* < 0.05). There was no significant difference between 50 mg/kg of larger-sized CuONPs (50 nm and 250 nm) and 100 mg/kg CuONP-250 nm or Cu^2+^ ions (*p* < 0.05) (Figure 4A). Generally, root Cu uptake was similar for Cu^2+^ ions and largest size of CuONPs–250 nm, unlike the smallest size of CuONPs–25 nm that had the highest root Cu uptake responses in soybeans (Figure 4A).

Several factors, including plant species, concentration used, root morphology, and soil properties, can influence Cu uptake and bioaccumulation in plants [65]. Phytochelatins and metallothionein, which are typical organic complexes that likely form in root cells, can enhance the retention of Cu at the soil–root interface [66]. Cu ions from NPs have been shown to decrease root length, water content, and dry biomass in lettuce. A significant accumulation of Cu in roots exposed to Cu/CuONPs (20–30 nm) was documented compared to CuSO_4_.5H_2_O in lettuce [67]. Previous reports have indicated the presence of a significant amount of Cu in plant roots exposed to CuONPs. Andreotti et al. [68] found differences in Cu translocation in two salt marsh plant species (*Halimione portulacoides* (L.) Aellen, and *Phragmites australis* (Cav.) Trin.). While Cu accumulated in the root of both plants, accumulation was significantly lower (4–10 times) when Cu was added as NPs. For *H. portulacoides,* no Cu translocation occurred in roots at 10 ppm NP (<50 nm) treatment, whereas for *P. australis*, Cu translocation occurred regardless of the type of Cu used (CuCl_2_ or CuONPs <50 nm) [68]. In a recent study using cowpea, Ogunkunle et al. [69] found a linear concentration response upon exposure to CuNPs (<25 nm and 60–80 nm) for Cu uptake by root, a finding consistent with our results. Deng et al. [70] found the highest Cu concentration in *Brassica rapa* var. Rosie treated with 600 mg/kg of CuONPs, with roots containing up to ~479 mg Cu/kg dry weight.

### 4.3. Stem Copper Uptake

ANOVA showed a significant effect of Cu compound type (Cu_type_; *p* < 0.0001), concentration (C; *p* < 0.0001), and the interaction term (Cu_type_ × C; *p* < 0.001) on stem Cu uptake in soybean (Table S2). Particle size- and concentration-dependent Cu uptake was observed in soybean stem upon exposure to CuONPs. The concentration–response curves for all CuONPs and Cu^2+^ ions were linear (Table 4). CuONP–25 nm treatment led to significantly greater Cu uptake in stem compared to the larger-sized CuONP and Cu^2+^ ion treatments, and this uptake increased nearly two-fold at 500 mg/kg treatment compared to 50 mg/kg treatment for all Cu compound types (Figure 4B). Stem Cu concentration increased to 7.3 mg/kg, on average, for CuONP–25 nm from the baseline of 3 mg/kg in the control. A similar stem Cu uptake was observed for plants treated with CuONP–250 nm compared to Cu^2+^ ion treatments at all concentrations tested (Figure 4B). Generally, stem Cu uptake was similar for Cu^2+^ ions and larger-sized (50 nm and 250 nm) CuONPs, unlike the smallest size of CuONPs–25 nm that had the highest stem Cu uptake responses in soybeans (Figure 4B).

Metal and metal oxide NPs can induce toxicity either by releasing toxic metal ions or by direct interaction with the cell [68,71]. Plants can absorb metals as dissolved or soluble ionic fractions, or as NPs themselves. Dimkpa et al. [58] observed Cu bioaccumulation in wheat stem upon exposure to 500 mg/kg CuONPs (<50 nm) and that soluble Cu from CuONPs was implicated in phytotoxicity. Consistent with our results, uptake of CuONPs by leaf fronds in *Landoltia punctata* (G. Mey) Les and D.J. Crawford was found to be more toxic compared to ionic Cu treatments [72]. Exposure to CuONPs (10–100 nm) and CuNPs (100–1000 nm) showed a reduction in root growth in lettuce and alfalfa, and increased stem Cu content in alfalfa [73].

### 4.4. Leaf Copper Uptake

Our ANOVA showed soybean leaf Cu concentration was significantly affected by Cu compound type (Cu_type_), concentration (C), and the interaction term (Cu_type_ × C) (*p* < 0.0001; Table S2). Furthermore, we found that leaf Cu uptake was particle size- and concentration-dependent. Similar to root Cu concentration, the concentration–response curves for the larger-sized CuONPs (CuONP–50 nm, CuONP–250 nm) and Cu^2+^ ions were deemed linear (Figure 4C and Table 4). Leaf Cu uptake with CuONP–25 nm treatments at 50, 100, 200, and 500 mg/kg concentrations increased by 3.9, 4.7, 5.7, and 6.0 times, respectively, compared to the control. Leaf Cu uptake was highest at 500 mg/kg CuONP–25 nm treatment but this was not significantly different from the 200 mg/kg CuONP-25 nm treatment. Moreover, the effects of larger-sized CuONPs (50 nm and 250 nm) were not significantly different between 50 and 500 mg/kg treatments (Figure 4C). Generally, leaf Cu uptake was similar for Cu^2+^ ions and larger-sized (50 nm and 250 nm) CuONPs, unlike the smallest size of CuONPs (25 nm) that had highest leaf Cu uptake responses in soybeans (Figure 4C).

ENPs must be absorbed by the root for uptake and accumulation in aerial plant parts. They enter vascular tissue (xylem) upon penetrating the cell wall and plasma membrane, translocating to stem, leaf, and ultimately to seed [23]. A linear relationship between Cu uptake/accumulation in different tissues and exposure concentrations of CuNPs was previously reported [23]. The pores in cell walls could be below 10 nm in diameter [23,74], which is much smaller than the size range that we tested for CuONPs. It was hypothesized that smaller-sized ENP aggregates can pass through the pores in cell walls, reaching the plasma membrane, unlike larger aggregates that may not be able to do so [75,76,77]. It is likely for ENPs to also create new pores upon cell surface interaction, enabling larger ENP internalization into plant tissues. However, cellular payloads, such as innate and foreign macromolecules (e.g., proteins, peptides), could actively transport in and out of the cell [78], and studies have documented that such larger molecules might transport via plasmodesmata, a roughly cylindrical channel reaching up to 40 nm in diameter [79,80,81].

Upon exposure to CuONPs of diameter 34–52 nm (100, 200, 500, 1000, and 2000 mg/L), Shi et al. reported reduced root length and NP accumulation in the roots and leaves in *Elsholtzia splendens* [82]. Cu bioaccumulation in cowpea leaves was also enhanced by CuNPs with sizes < 25 nm and 60–80 nm; however, increasing exposure concentrations led to decreased translocation [69], suggesting a threshold for NP uptake and translocation in plants. Deng et al. most recently reported that the leaf Cu accumulation pattern of *Brassica rapa* treated with CuONPs (75, 150, 300, and 600 mg Cu/kg soil) was dependent on particle size and plant phenotype [70]. Our findings suggest that CuONPs with a small size (25 nm) could be more favored for cellular entry and translocation compared to larger-sized CuONPs, promoting leaf Cu uptake in soybeans. Despite being bioavailable in the dissolved form, Cu^2+^ ions showed an overall lower uptake in different plant parts compared to the small-sized CuONPs–25 nm, suggesting a different mode of uptake of CuONPs compared to Cu^2+^ ions. In addition, the leaves in soybean plants fall off after ripening or before harvesting. Therefore, biofortification using leaves as a source for CuONPs (Figure 4C) may affect the microbial decomposition of soybean residues and warrants further investigation into its agronomic importance.

### 4.5. Seed Copper Uptake

Our analysis showed that the effect of Cu compound type (Cu_type_; *p* < 0.0001), concentration (C; *p* < 0.0001), and the interaction term (Cu_type_ × C; *p* < 0.01) were statistically significant for seed Cu uptake (Table S2). The findings generally show particle size- and concentration-dependent seed Cu uptake in soybeans upon exposure to CuONPs, and plants exposed to CuONP–25 nm typically showed greater Cu uptake compared to the larger-sized CuONPs (50 nm and 250 nm) or Cu^2+^ ions at most concentrations tested—a result consistent with the root, stem, and leaf Cu uptake (Figure 4D). At 500 mg/kg CuONP–25 nm treatment, the highest seed Cu uptake was observed; this was, on average, 1.8 times higher (6.55 mg/kg) than the control. The concentration–response curves for CuONPs and Cu^2+^ ions were deemed linear for seed Cu uptake, similar to other organs examined. Difference in seed Cu uptake at the 50 mg/kg CuONP–25 nm treatment was not statistically significant from the 50 mg/kg CuONP–50 nm, 100 mg/kg CuONP–250 nm, or 50 mg/kg Cu^2+^ ions treatments (*p* > 0.05). Analogous to Cu uptake in soybean stem, average Cu uptake in soybean seeds was not significantly different between the CuONP–250 nm and Cu^2+^ ions treatments at comparable concentrations. Furthermore, there was no statistical significance in seed Cu uptake between CuONP–50 nm and Cu^2+^ ions at 50, 200, and 500 mg/kg treatments (*p* *>* 0.05; Figure 4D). Generally, seed Cu uptake was similar for Cu^2+^ ions and larger-sized (50 nm and 250 nm) CuONPs, unlike the smallest-sized CuONPs–25 nm that had the highest seed Cu uptake responses in soybean (Figure 4D).

Potential aggregation of CuONP–250 nm at higher concentrations may decrease metal bioavailability, thus reducing metal uptake and toxicity. In our study, total Cu concentrations differed in the order of roots > leaves > stem > seeds (Figure 4), suggesting that the organs farthest away from the root-soil system had the lowest total Cu (i.e., seeds) and the root that is in direct contact with the soil had the highest total Cu levels. Wang et al. also showed metallic NPs’ phloem-based translocation from leaves to other parts of plant. CuONP–25 nm enabled greater Cu translocation to seeds [83], probably due to their smaller size that facilitated easier passage through the cell wall. NPs with a smaller particle size can increase Cu bioavailability even at low concentration, which may be due to increasing surface area of the smaller size NPs [68]. However, CuONPs with a larger particle size can form more aggregates with reduced surface area and thus decrease Cu bioavailability, especially in the seed. Seeds of cowpea accumulated a significant level of Cu compared to the control, and the highest Cu level was related to CuNPs < 25 nm and 60–80 nm, at 500 mg/kg and 1000 mg/kg, respectively [69]. Cu/CuNP treatments altered nutritional quality compared to the control as demonstrated by more Cu, S, and Al, but less Mg, Ca, P, and Mn, in lettuce [67].

### 4.6. Soil pH and EC

**Figure 5:**
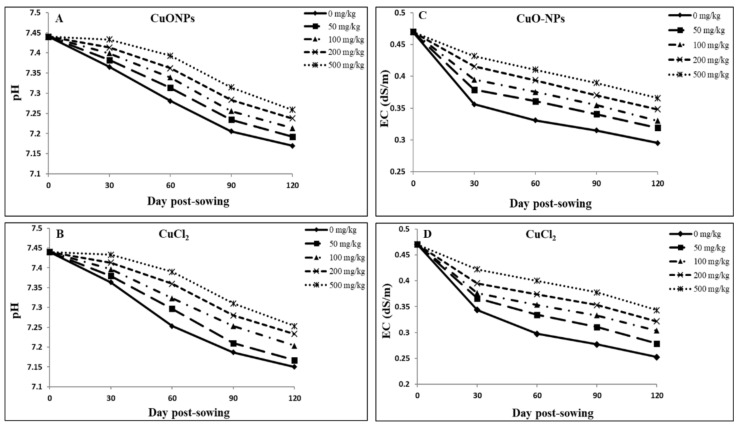
Changes in soil pH (**A**,**B**) and electrical conductivity (EC) (**C**,**D**) of soil amended with CuONPs (**A**,**C**) and CuCl_2_ (**B**,**D**) during the full growth period of 120 days in soybean. The data are averaged for all three types of CuONPs as they were similar among the NP types.

Figure 5A,B demonstrate post-harvest soil pH change in soil amended with various concentrations of different sized CuONPs and CuCl_2_. Overall, the trends are similar, with the higher concentrations eliciting lower pH change than at lower concentrations for both the NPs and ions of Cu.

Normal root activities, including proton secretion, microbial activity at the soil–root interface, and the potential release of root exudates, can contribute to soil acidification to some degree [61,84]. Exposure to both compound types caused similar soil pH changes, suggesting similar mechanisms might be at play, leading to increased H^+^ ions concentrations. However, the presence of different organic and inorganic compounds capable of complexing Cu and altering its solubility could influence overall Cu bioavailability, independent of pH [84]. Our findings indicate that during the growth period, the soil pH reduced but remained alkaline (Figure 5A,B). At low pH, higher concentrations of NPs are more phytotoxic owing to potential for more dissolution of metals [85]. Accordingly, as CuONP concentration increased, the bioavailable Cu increased with decreasing pH. Shi et al. [82] found CuONP dissolution was promoted inside the cell due to decreased cellular pH.

In the present study, with reducing pH as Cu concentration increased in soil, a greater Cu uptake was associated with lower root growth (Table 3 and Table 5), which could lead to decreased plant growth and yield in soybean plants [47]. Figure 5B shows the pH changes for CuCl_2_-amended soil at different concentrations, which were similar to the pH changes documented for CuONPs (Figure 5A).

Cu is a low-mobility element in soil but can form very intense chelates [66,86]. Potential toxicity of metal oxide NPs might be due to the dissolution and release of metal ions. Dissolution can be affected by environmental conditions, for example, pH and EC, to which ENPs are subjected upon release into the environment [68]. Soil pH can affect metal bioavailability and phytotoxicity of NPs [48]. Generally, an alkaline condition promotes NPs aggregation, while an acidic environment could trigger metal and oxide NPs’ dissolution, transforming them into ionic species [87,88]. Additionally, in alkaline pH, aggregation of NPs can alter nano-specific attributes, and their dissolution into ionic forms could decrease. However, with a pH change, the NPs can also disaggregate and return to a previous stable state. Likewise, NP reactivity and toxicity can be modified with a minor change in surface charge and particle size [89], and this can also change as a function of media chemistry [90,91,92].

Figure 5C,D show changes in post-harvest soil electrical conductivity (EC) under different sized CuONP and CuCl_2_ treatments. Overall, the trends are similar, with higher concentrations eliciting lower EC changes than at lower concentrations for both the NPs and Cu^2+^ ions. These results indicate lower root activities at higher concentration treatments likely leading to a lower root exudate release into the soil–root interface and thus a lower change in EC values. Furthermore, similar soil pH and EC changes with CuONPs and Cu^2+^ ions suggest that soil-root physicochemical behavior may not be a major player driving Cu uptake in soybeans. More detailed studies are warranted to determine if the minor soil pH and EC changes could have driven disparate Cu uptake in soybean plants under CuONP and Cu^2+^ ion treatments.

### 4.7. Implications for Human Nutrition

The National Academies of Sciences, Engineering and Medicine’s Food and Nutrition Board (FNB) has developed the Dietary Reference Intakes (DRIs) for Cu and other nutrients for human intake recommendations [51]. The DRI offers a set of reference values used for planning and assessing nutrient intakes for healthy people. Recommended Dietary Allowance (RDA) is an average daily intake considered sufficient to meet the nutrient requirements of (almost) all (97–98%) healthy individuals and is typically employed in planning diets that are nutritionally adequate for individuals [51]. The U.S. Food and Drug Administration (FDA) has developed Daily Values (DVs) to assist consumers with comparing the nutrient contents in foods and dietary supplements within the context of a total diet. The FDA required manufacturers to use these new labels starting in January 2020, but companies with annual sales of less than $10 million may continue to use the old labels that list a DV of 2 mg (2000 µg) for Cu until January 2021 [93,94]. The FDA does not require food labels to list Cu content unless it has been added to the food. Foods that offer ≥20% of the DV are considered to be high sources of a nutrient, but foods providing lower percentages of the DV may also contribute to a healthy diet [51].

Our results of soybean seed Cu concentrations following different sized CuONP (25 nm, 50 nm, 250 nm) treatments at variable soil Cu concentrations (50–500 mg/kg soil) demonstrated the potential for significant improvement in seed Cu uptake (Figure 4D and Table 6) with DV values in the range 44.0–73.0%. For Cu^2+^ ions, the DV values were in the range of 44.2–67.0%. The highest DV values were recorded for the smallest-sized CuONPs–25 nm (DV = 47.5–73.0%), followed by CuONPs–50 nm (DV = 45.6–69.0%) and CuONPs–250 nm (DV = 44.0–65.0%). The DV values for ionic Cu^2+^ mirrored that of the largest-sized CuONPs–250 nm (Table 6).

Comparing soybean seed Cu concentrations with chickpea seeds, a legume considered a good source of nutrients and phenolic compounds (e.g., polyphenols, isoflavones) with antioxidative potential to reduce oxidative effects with evidence supporting its consumption in prevention and management of diabetes and obesity [95], we found that our soybean seeds had 1.38–2.30 fold greater Cu concentrations than chickpea seeds per serving of ½ cup (100 g or 3.5 ounces) (Table 6). These results suggest that soil amendment by CuONPs, specifically by the smallest-sized CuONPs–25 nm, could significantly improve nutritional Cu value in soybean seeds, and is found to be a better source of nutritional Cu compared to several other food items that provide nutritional Cu, including: Atlantic salmon (wild, cooked), avocado, asparagus, cream of wheat, dried figs, ground turkey, Greek yogurt, non-fat milk, pasta, sesame seeds, and whole wheat (Table 6) [51]. Furthermore, higher Cu soil amendment that led to greater seed Cu concentrations in soybean is comparable to or greater than the food items which often provide higher DV of Cu, including cooked mushrooms (1/2 cup), cashew nuts (dry roasted, 1 ounce), cooked crab (Dungeness, 3 ounces), sunflower seed kernels (1/4 cup), simmered turkey giblets (3 ounces), dark chocolate (1 ounce), and raw tofu (½ cup) [51]. Moreover, our soybean seeds were rated “Good” to “Very Good” according to the “World’s Healthiest Foods” rating, based on the DV values (Table 6) [54].

**Figure 6:**
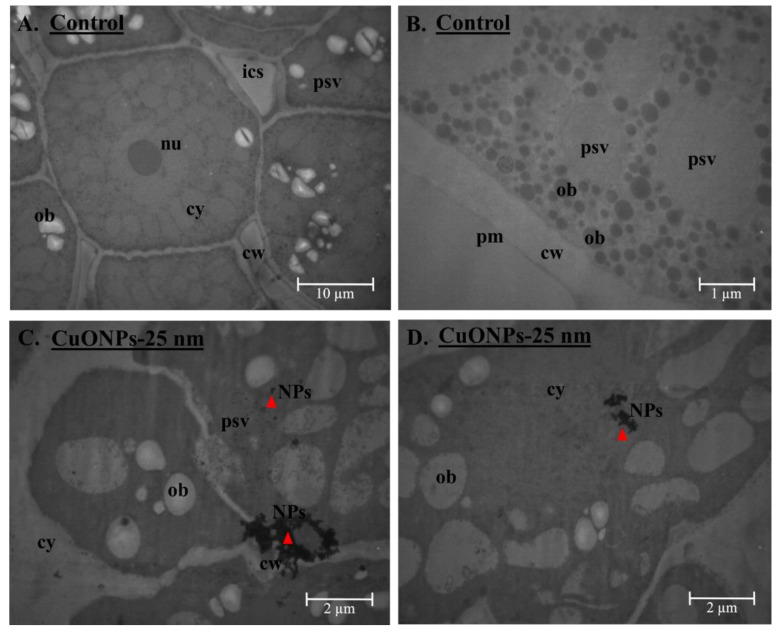
TEM analysis of ultrastructure of soybean seed embryo with CuONPs–25 nm treatment at 500 mg/kg soil (**C**,**D**) and compared with control seeds (no nanoparticles) (**A**,**B**). Electron-dense metal aggregates are clearly visible within the cell wall (cw)/plasma membrane (pm), including within the cytoplasm (cy) and/or protein storage vacuoles (psv) for the seeds with CuONPs–25 nm treatment at 500 mg/kg soil (**C**,**D**). nu = nucleus, ics = intracellular space, ob = oil bodies, NPs = nanoparticles (red triangle).

Because we found evidence of CuONPs within the cell wall, cell membrane, and protein storage vacuoles within the cytoplasm of the soybean seed embryo using electron microscopy for CuONPs–25 nm treatment (Figure 6), it is paramount to understand the potential toxicity of CuONPs in humans upon consumption of soybean seeds and oil. Future research should address what risk, if any, CuONP may pose to humans before such Cu-fortified soybean seeds are recommended for daily human consumption to address Cu deficiency and associated illnesses, globally.

## 5. Conclusions

Utilization of CuONPs in modern agriculture as a novel fertilizer requires an understanding of their uptake, translocation and toxicity in plants. This study showed particle size- and concentration-dependent effects of CuONPs on Cu uptake and biodistribution in root, stem, leaf and seed in soybean grown in soil for a full lifecycle of 120 days. Our observations of the altered root architecture were dependent on the Cu compound types, Cu concentrations, and their interactions. We found that the concentration–response curves for all three sizes CuONPs, including the Cu^2+^ ions, were linear. CuONPs and Cu^2+^ ions showed inhibitory effects on root growth and development. Overall, the responses of soybean to the smallest size CuONPs–25 nm were greater for all parameters, compared to the two larger-sized CuONPs (50 nm and 250 nm) or Cu^2+^ ions, tested. However, similar soil pH and EC changes with CuONPs and Cu^2+^ ions suggest that soil-root physicochemical attributes may not be a major player driving Cu uptake in soybean. Plant bioaccumulation of Cu in different parts followed the order of root > leaf > stem > seed. Limited root growth could result in reduced water and soil nutrient utilization, potentially inhibiting plant growth and yield, and this is consistent with our companion study showing particle size- and concentration-dependent reduction in seed yield in soybean with the (same) CuONP treatments (Figure S1) [47]. Although the root architecture was reduced, the smallest-sized CuONPs-25 nm led to increased seed Cu uptake compared to the larger-sized CuONPs or Cu^2+^ ions. Furthermore, our results suggest that soil amendment with CuONPs—more importantly, with the smallest-sized CuONPs–25 nm—could significantly improve nutritional Cu value in soybean seeds as demonstrated by % Daily Values, which are rated “Good” to “Very Good” as per the “World’s Healthiest Foods” rating. However, it is imperative to understand the potential toxicity and risk upon consumption of soybean seeds containing Cu-based NPs before CuONP-fortified soybean seeds are recommended for daily human consumption while addressing global Cu deficiency and associated illnesses in humans.

## Figures and Tables

**Table 1:** Summary of the structural parameters of CuONPs obtained from the Rietveld analysis of the XRD patterns.

Sample	S1	S2	S3
Structure	Monoclinic	Monoclinic	Monoclinic
Space group	*C2*/*c*	*C2*/*c*	*C2*/*c*
Lattice parameters			
*a* (Å)	4.68369	4.68678	4.68469
*b* (Å)	3.42223	3.42659	3.42223
*c* (Å)	5.12774	5.13265	5.12964
α (°)	90.00	90.00	90.00
β (°)	99.37073	99.4	99.49814
γ (°)	90.00	90.00	90.00
*Unit cell Volume* (Å)^3^	81.094	81.321	81.111
*Particle size* (nm)	25	50	250
*Reliability factors*			
*Weighted profile factor* (R_wp_)	13.3	12.1	12.9
*Profile factor (R_p_)*	16.1	14.7	17.4
*Expected R-factor* (R_exp_)	10.41	9.78	10.87
*Bragg R-factor* (R_Bragg_)	1.65	1.29	1.10
*R-factor (R_F_)*	1.16	1.01	0.843
*Chi squared* (χ^2^)	1.85	1.80	1.90

**Table 2:** Statistical analysis (ANOVA) of root morphological parameters for soybean grown in soil amended with different copper (Cu) compound types and concentrations.

Source of Variation		Root Dry Weight	Root Length	Root Volume	Root Area	Root Density
Copper compounds type (Cu_type_)	MS	7.48	128.74	87.02	1315	0.0053
	df	3	3	3	3	3
	*F*	32.54	28.77	12.66	31.40	8.93
	*P*	<0.0001	<0.0001	<0.0001	<0.0001	0.0001
Compounds concentration (C)	MS	21.23	366	294.7	4046	0.0096
	df	4	4	4	4	4
	*F*	92.27	81.79	42.87	96.60	16.27
	*p*	<0.0001	<0.0001	<0.0001	<0.0001	<0.0001
Cu_type_ × C	MS	0.55	14.28	13.39	155.2	0.0005
	df	12	12	12	12	12
	*F*	2.40	3.19	1.95	3.71	0.95
	*p*	0.0188	0.0028	0.0572	0.0009	0.5139

**Table 3:** Effects of CuONPs and CuCl_2_ on mean root dry weight and mean root length in soybean. Means with similar letters denote no significant difference between the treatments according to the Fisher’s LSD test (*p* ≤ 0.05).

**Concentration (mg/kg soil)**	**Mean Root Dry Weight (g/plant)**				**Mean Root Length (cm)**			
	CuONPs			CuCl_2_	CuONPs			CuCl_2_
	25 nm	50 nm	250 nm		25 nm	50 nm	250 nm	
0	5.45 ^a^	5.45 ^a^	5.45 ^a^	5.45 ^a^	38.60 ^a^	38.60 ^a^	38.60 ^a^	38.60 ^a^
50	3.02 ^f^	4.08 ^bc^	4.42 ^bc^	4.81 ^ab^	34.22 ^bc^	34.22 ^bc^	35.16 ^abc^	37.83 ^a^
100	2.20 ^ghi^	3.27 ^def^	4.02 ^bcd^	4.30 ^bc^	26.50 ^ef^	32.53 ^cd^	34.01 ^bc^	37 ^ab^
200	1.53 ^ij^	2.63 ^fgh^	2.99 ^fg^	3.83 ^cde^	22.50 ^g^	26.50 ^ef^	29.83 ^de^	32.33 ^cd^
500	0.94 ^j^	1.67 ^ij^	2.05 ^hi^	3.16 ^ef^	18.83 ^h^	25.03 ^fg^	26.05 ^f^	30 ^d^

**Table 4:** Coefficient of determination (R-squared value) from the linear regression for various parameters tested for different sized CuONP and CuCl_2_ treatments in soil-grown soybeans.

**Parameters**	**R-Squared**			
	CuONP–25 nm	CuONP–50 nm	CuONP–250 nm	CuCl_2_
Root dry weight	0.60 (NL)	0.78 (L)	0.87 (L)	0.85 (L)
Root length	0.75 (L)	0.76 (L)	0.89 (L)	0.88 (L)
Root volume	0.68 (L)	0.65 (L)	0.43 (NL)	0.67 (L)
Root area	0.72(L)	0.71 (L)	0.72 (L)	0.84 (L)
Root Cu uptake	0.64 (NL)	0.90 (L)	0.97 (L)	0.99 (L)
Stem Cu uptake	0.96 (L)	0.90 (L)	0.85 (L)	0.79 (L)
Leaf Cu uptake	0.55 (NL)	0.74 (L)	0.87 (L)	0.72 (L)
Seed Cu uptake	0.85 (L)	0.87 (L)	0.89 (L)	0.90 (L)

The concentration–response curve was deemed linear if the R-squared value was 65% or higher. “L” denotes linear, and “NL” denotes nonlinear concentration–response relationship.

**Table 5:** Effect of CuONPs and CuCl_2_ on root volume and root area of soybeans. Means with similar letters denote no significant difference between the treatments according to the Fisher’s LSD test (*p* ≤ 0.05).

**Concentration (mg/kg)**	**Mean Root Volume (cm^3^)**				**Mean Root Area (cm^2^)**			
	CuONPs			CuCl_2_	CuONPs			CuCl_2_
	25	50	250		25	50	250	
0	33.34 ^a^	33.34 ^a^	33.34 ^a^	33.34 ^a^	126.94 ^a^	126.94 ^a^	126.94 ^a^	126.94 ^a^
50	28.16 ^bc^	28.66 ^bc^	28.83 ^bc^	30.83 ^ab^	110.01 ^c^	110.89 ^bc^	112.84 ^bc^	121 ^ab^
100	22.66 ^efg^	23.33 ^defg^	25.16 ^cdef^	27.50 ^bcd^	86.73 ^fgh^	97.44 ^de^	103.45 ^cd^	112.98 ^bc^
200	17 ^hi^	21 ^fgh^	25 ^cdef^	27.16 ^bcd^	69.27 ^ij^	83.60 ^gh^	96.70 ^def^	104.95 ^cd^
500	16 ^i^	19.33 ^ghi^	24.83 ^cdef^	25.48 ^cde^	61.21 ^j^	77.96 ^hi^	90.13 ^efg^	97.86 ^de^

**Table 6:** Cu concentrations in soybean seeds compared to Recommended Daily Allowances (RDA), Cu concentrations in chickpea seeds, and % Daily Values (DV) based on the U.S. Food and Drug Administration (FDA) recommendations for adults and children aged 4 years and older [94].

Experimental				Cu Concentration in Chickpea Seeds (µg Cu/100 g Seed)	Fold (×) Higher than Chickpea Seed Cu Concentration (per Serving of ½ Cup)	Recommended Dietary Allowances (RDA) for Cu Intake for Adults and Children Aged 4 Years and Older (µg)	% Daily Values (DV) *	World’s Healthiest Foods Rating ^#^	Food Items that Provide Lower Cu than Our Soybean Seeds ^£^
Treatment Types	Concentration Applied (mg CuONPs/kg Soil)	Mg Total Cu/kg Soybean Seed	µg Total Cu/100 g Soybean Seed						
CuONPs-25 nm	50	4.28	428	289	×1.48	900	47.5	Good	Atlantic salmon (wild, cooked), avocado, asparagus, cream of wheat, dried figs, ground turkey, Greek yogurt, non-fat milk, pasta, sesame seeds, whole wheat
	100	5.00	500		×1.73		55.5	Very Good	
	200	5.80	580		×2.00		64.4	Very Good	
	500	6.55	655		×2.30		73.0	Very Good	
CuONPs-50 nm	50	4.11	411		×1.42		45.6	Good	
	100	4.74	474		×1.64		53.0	Very Good	
	200	5.48	548		×1.90		61.0	Very Good	
	500	6.21	621		×2.15		69.0	Very Good	
CuONPs-250 nm	50	3.94	394		×1.36		44.0	Good	
	100	4.4	440		×1.52		49.0	Good	
	200	5.19	519		×1.80		58.0	Very Good	
	500	5.81	581		×2.01		65.0	Very Good	
CuCl_2_	50	3.98	398		×1.38		44.2	Good	
	100	4.41	441		×1.53		49.0	Good	
	200	5.31	531		×1.84		59.0	Very Good	
	500	6.03	603		×2.10		67.0	Very Good	
Control	0	3.62	362		×1.25		40.2	Good	

An amount of 100 g of soybean seeds is assumed to be equivalent to 100 g of chickpea seeds per serving of ½ cup, which is equivalent to 3.5 ounces. % DV = % RDA. * The DV for copper on the new Nutrition Facts and Supplement Facts labels and used for the values in Table 4 is 0.9 mg (900 ug) for adults and children aged 4 years and older (US FDA, 2016). Foods providing 20% or more of the DV are considered to be high sources of a nutrient. ^#^ The “World’s Healthiest Foods” rating is based on a simple rule: Excellent, if DV ≥ 75%; Very Good, if DV ≥ 50%; Good, if DV ≥ 25% [54]. ^£^ Food items for which Cu levels are compared are adopted from the U.S. Department of Agriculture, Agricultural Research Service’s FoodData Central [53].

## Supplementary Materials

The following are available online at https://www.mdpi.com/2223-7747/9/10/1326/s1, Figure S1: Variation in dry weight in root, stem, leaf and seed, Table S1: soil physiochemical properties, Table S2: statistical analysis of Cu uptake in various tissues.
