# Supplementary material for: Root System Architecture, Copper Uptake and Tissue Distribution in Soybean (Glycine max (L.) Merr.) Grown in Copper Oxide Nanoparticle (CuONP)-Amended Soil and Implications for Human Nutrition
Source: Plants (Basel). 2020 Oct 8;9(10):1326. doi: 10.3390/plants9101326 (PMC7600329; doi:10.3390/plants9101326)
Supplement: Supplementary file 1 [file plants-09-01326-s001.pdf]

plants

MDPI

**Supplementary Information**

# Root system architecture, copper uptake and tissue distribution in soybean (*Glycine max* (L.) Merr.) grown in copper oxide nanoparticle (CuONP)-amended soil and implications for human nutrition

[1]Yusefi-Tanha, Elham; [1*]Fallah, Sina; [2]Rostamnejadi, Ali; [3*]Pokhrel, Lok Raj

[1]Department of Agronomy, Faculty of Agriculture, Shahrekord University, Shahrekord, Iran
[2] Department of Electroceramics and Electrical Engineering, Malek Ashtar University of Technology, Iran. Email: rostamnejadi@mut-es.ac.ir.
[3]Department of Public Health, The Brody School of Medicine, Department of Health Education and Promotion, College of Health and Human Performance, East Carolina University, Greenville, NC, USA.

*Corresponding authors: LR Pokhrel, Phone: 252-737-5587; Email: pokhrell18@ecu.edu.
S Fallah, Phone: +983832324428; Email: fallah-s@sku.ac.ir.

**Table S1. Physicochemical properties of the soil used in the experiment.**

| pH | EC (dS/m) | Organic carbon (g/kg) | Total N (g/kg) | Available P (mg/kg) | Available K (mg/kg) | Cu (mg/kg) | Zn (mg/kg) | Fe (mg/kg) | Mn (mg/kg) |
|---|---|---|---|---|---|---|---|---|---|
| 7.44 | 0.47 | 9.24 | 0.88 | 11.7 | 405 | 0.538 | 0.892 | 5.12 | 6.85 |

**Table S2. Statistical analysis (ANOVA) of copper (Cu) uptake in different tissues (root, stem, leaf, and seed) for soybean grown in soil-amended with different Cu compounds types and concentrations.**

| Source of variation | | Cu concentration | | | |
|---|---|---|---|---|---|
| | | **Root** | **Stem** | **Leaf** | **Seed** |
| **Copper compounds types ($Cu_{type}$)** | MS | 60.36 | 2.79 | 9.08 | 0.61 |
| | df | 3 | 3 | 3 | 3 |
| | *F* | 419 | 20.74 | 104 | 32.17 |
| | *P* | <.0001 | <.0001 | <.0001 | <.0001 |
| **Compounds concentration (C)** | MS | 414.73 | 17.66 | 61.94 | 12.53 |
| | df | 4 | 4 | 4 | 4 |
| | *F* | 2878 | 131.2 | 709.5 | 657.8 |
| | *p* | <.0001 | <.0001 | <.0001 | <.0001 |
| **$Cu_{type}$ × C** | MS | 11.61 | 0.53 | 1.04 | 0.052 |
| | df | 12 | 12 | 12 | 12 |
| | *F* | 80.62 | 3.98 | 11.92 | 2.74 |
| | *p* | <.0001 | 0.0005 | <.0001 | <0.01 |

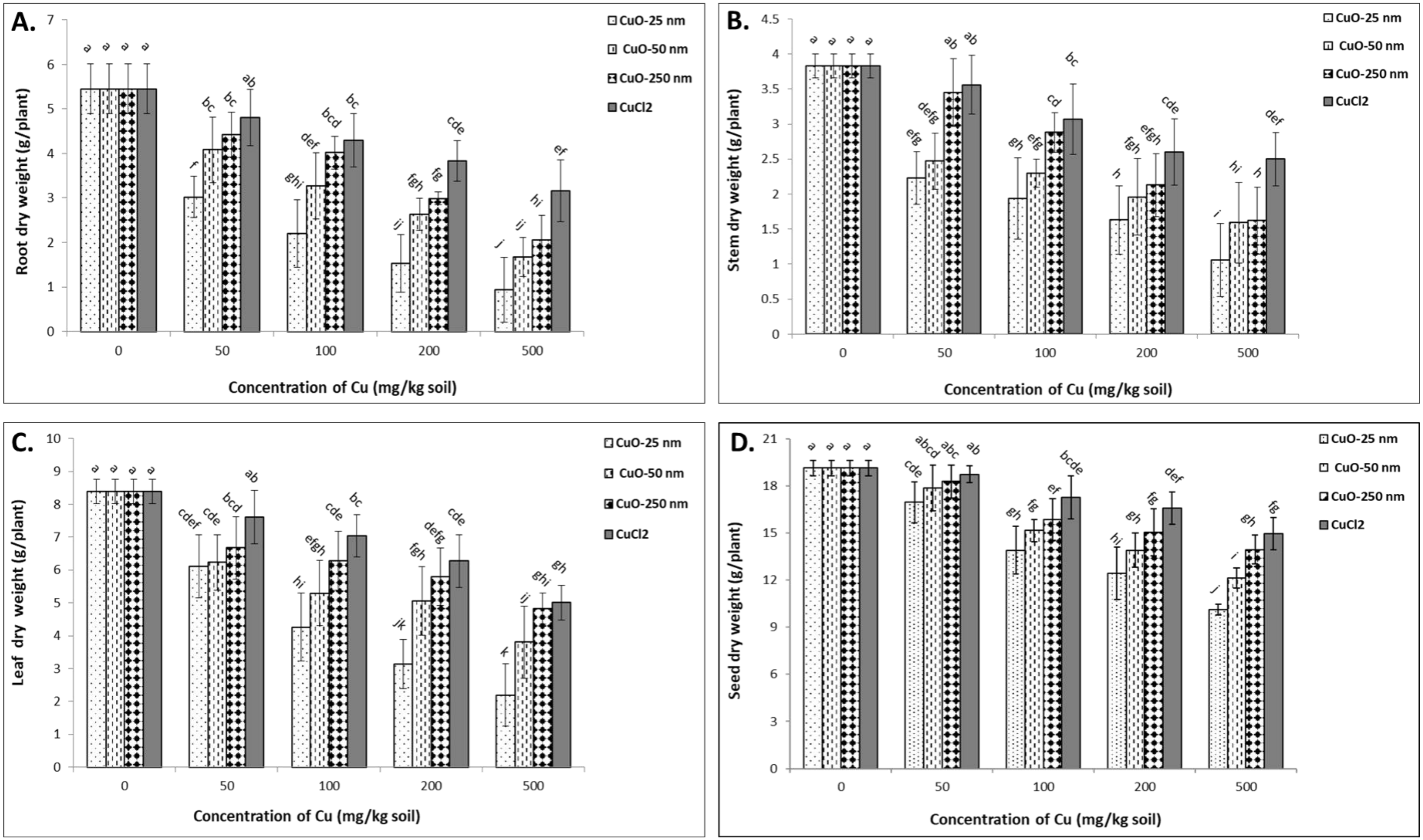


**Fig. S1**. Variation in root dry weight (A), stem dry weight (B), leaf dry weith (C) and seed dry weight (yield) in soybean exposed to different types of copper compounds (CuONP–25 nm, CuONP–50 nm, CuONP–250 nm, and $Cu^{2+}$ ions) as a function of concentrations (0, 50, 100, 200, 500 mg/kg-soil). The biomass responses of different plant parts show a dependency on particle size and concentration of CuONPs and $Cu^{2+}$ ions. Figure D is adopted from our companion paper (see Figure 2 in Ref. 47). Same letter above the bars indicates not significant difference between the treatments according to the Fisher's LSD test at $p \leq 0.05$.
